# Noninvasive Early Detection of Systemic Inflammatory Response Syndrome of COVID-19 Inpatients Using a Piezoelectric Respiratory Rates Sensor

**DOI:** 10.3390/s24227100

**Published:** 2024-11-05

**Authors:** Tsuyoshi Kobayashi, Takemi Matsui, Isamu Sugita, Norihiro Tateda, Shohei Sato, Kenichi Hashimoto, Masei Suda

**Affiliations:** 1 Graduate School of Systems Design, Tokyo Metropolitan University, Tokyo 191-0065, Japan; tsuyoshi.kobayashi@konicaminolta.com (T.K.);; 2 Konica Minolta, Inc., Tokyo 192-8505, Japan; 3 Department of Rehabilitation, Suwa Central Hospital, Nagano 391-8503, Japan; 4 Department of General Medicine, National Defense Medical College, Saitama 359-8513, Japan; 5 Department of Rheumatology, Suwa Central Hospital, Nagano 391-0011, Japan; 6 Immuno-Rheumatology Center, St. Luke’s International Hospital, Tokyo 104-8560, Japan

**Keywords:** systemic inflammatory respiratory syndrome, without blood sampling, noninvasive, respiratory rate, piezoelectric respiratory sensor, COVID-19

## Abstract

In 2020, 20% of patients with COVID-19 developed severe complications, including life-threatening pneumonia with systemic inflammatory response syndrome (SIRS). We developed a preliminary SIRS monitor that does not require blood sampling, is noninvasive, and can collect data 24 h per day. The proposed monitor comprises a piezoelectric respiratory sensor located beneath the patient’s mattress and a fingertip pulse sensor that determines ultra-high accuracy respiratory rate (mode of a 40-min frequency distribution of respiratory rates (M40FD-RR)). We assessed the clinical performance of the M40FD-RR preliminary SIRS monitor in 29 patients (12 female, 17 male, aged 15–90 years) hospitalized at Suwa Central Hospital with COVID-19, which was confirmed by a positive polymerase chain reaction test. SIRS was evaluated by logistic regression analysis using M40FD-RR, heart rate, age, and sex as explanatory variables. We compared the results of 109 examinations of 29 COVID-19 inpatients with SIRS against those determined by the proposed monitor. The proposed monitor achieved 75% sensitivity and 83% negative predictive value, making it a promising candidate for future 24 h noninvasive preliminary SIRS tests.

## 1. Introduction

In 2020, 20% of patients with COVID-19 developed severe complications, including life-threatening pneumonia with systemic inflammatory response syndrome (SIRS) [1,2]. SIRS indices [3] are index values for determining SIRS, which were initially proposed in 1992 and still in use today. Similarly, the q-SOFA [4], proposed in 2016, is also widely used as an early indicator for sepsis, which is a serious complication of infectious diseases. Recent research suggests that the SIRS indices have higher sensitivity for predicting infectious diseases and are considered particularly useful for screening [5]. We have previously reported on vital signs-based infection screening methods using SIRS indices [6,7].

In this paper, we describe a SIRS monitor for use in COVID-19 patients based on high-accuracy respiratory rate (RR) monitoring achieved through a piezoelectric respiratory sensor-derived mode of frequency distribution of the breaths over 40 min (M40FD-RR) and heart rate (HR), respectively. It has been reported that respiratory abnormalities often appear as a precursor to worsening COVID-19 symptoms [8]. Conventional RR monitors used in the intensive care unit, such as capnography or thoracic impedance pneumography, place a burden on patients. This article describes a new RR monitor based on a piezoelectric sensor that does not require direct contact with the body and showed promising laboratory test results [9]. Using a piezoelectric sensor at night in clinical settings with fewer body movements, Tanaka et al. [10] achieved higher RR accuracy than with thoracic impedance pneumography using visual RR counts by nurses. To achieve high RR accuracy all day, including during the day when more body movements occur, we proposed a new algorithm-derived high-accuracy RR called M40FD-RR.

To promote early testing for SIRS by medical professionals, we developed a preliminary SIRS monitor using vital signs, including M40FD-RR, that does not require blood sampling.

## 2. Materials and Methods

### 2.1. System Configuration of a 40-min Frequency Distribution of Respiratory Rates (M40FD-RR) SIRS Monitor

#### 2.1.1. Hardware Composition

**Figure 1:**
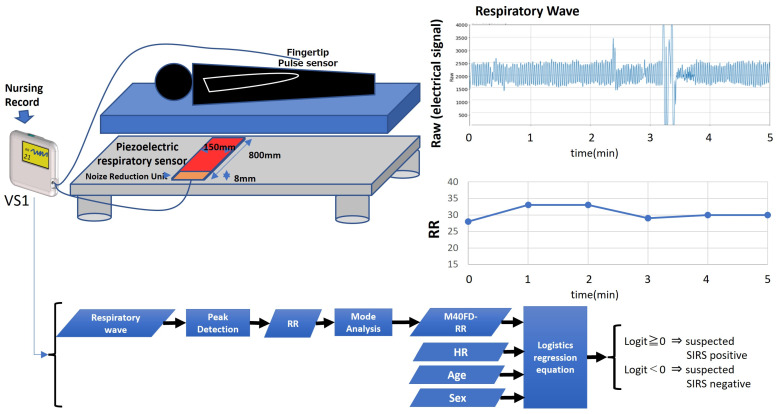
The frequency distribution of respiratory rates was recorded over 40 min (M40FD-RR) using the systemic inflammatory response syndrome (SIRS) early detection monitor. An accurate respiratory rate (RR) determination algorithm, M40FD-RR, can be used even with artifacts, including body movements. The M40FD-RR SIRS monitor consists of a piezoelectric respiratory sensor located beneath the patient’s mattress and a fingertip pulse sensor for heart rate (HR). RR is measured by the peak interval of the respiratory wave derived from the piezoelectric respiratory sensor. Logistic regression analysis was used to identify patients suspected to be SIRS-positive using M40FD-RR, HR, age, and sex as explanatory variables (logit score ≥ 0 suspected as SIRS-positive; logit score < 0 suspected as SIRS-negative).

Figure 1 shows the system configuration of a preliminary SIRS monitor and its data processing algorithm. The proposed system comprises a prototype piezoelectric respiratory sensor (Konica Minolta Inc., Tokyo, Japan) located beneath the patient’s mattress, a disposable fingertip pulse probe (TL-271 Series, Nihon Kohden, Tokyo, Japan), a monitor (VS1 [11], Konica Minolta Inc., Tokyo, Japan) that indicates RR, fingertip pulse monitor-derived heart rate, and nursing records.

A piezoelectric respiratory sensor measures the dynamic pressure change induced by respiratory and non-respiratory body movements as an electrical signal to obtain the body movement wave. The respiratory wave is separated from the body movement wave by using a bandpass filter (bandpass from 0.1 to 0.7 Hz) to attenuate non-respiratory body movements with frequencies lower than 0.1 Hz and artifacts with frequencies higher than 0.7 Hz. The bandpass filter monitors tachypnea-induced respiratory waves up to 42 BPM. It also monitors bradypnea from 6 to 10 BPM.

#### 2.1.2. Data Processing Algorithm of the M40FD-RR SIRS Monitor

**Figure 2:**
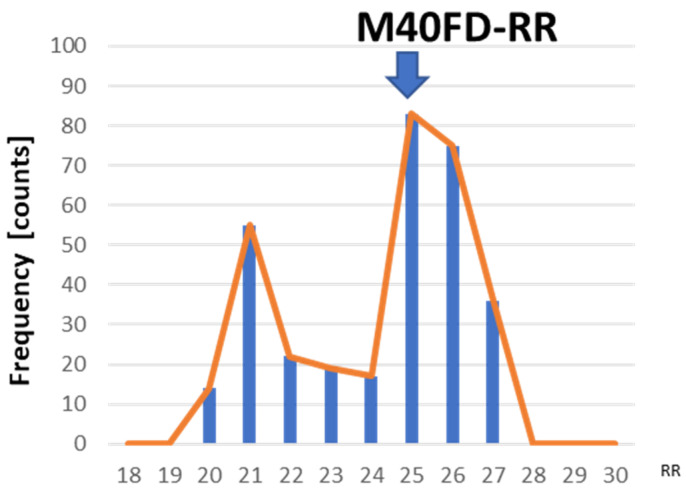
Frequency distribution of approximately 500 RR values recorded over 40 min. M40FD-RR is determined from the mode of this frequency distribution.

The M40FD-RR SIRS monitor obtains the respiratory wave from a piezoelectric respiratory sensor (Figure 1). The RR is calculated from the peak-to-peak respiratory wave and averaged over 90 s (Figure 1). To determine the RR with high precision, we proposed the mode of frequency distribution of breaths over 40 min (M40FD-RR). Approximately 500 RR values recorded over 40 min were used to determine M40FD-RR (Figure 2).

The M40FD-RR SIRS monitor uses logistic regression analysis to separate SIRS-positive and negative cases. Logistics analysis is an optimal method for classifying subjects into two categories and is flexible for handling both continuous variables (RR, HR, etc.) and categorical variables (sex, age, etc.). Logistics analysis is often used as an easy-to-understand learning method in the medical field, such as when predicting the risk of a patient developing a certain disease. The logistic regression model (1) and logit score (2) can be expressed by the following formula: (objective variable *Y*: 0/1, probability of *Y* = 1: *P*(*Y* = 1), explanatory variables *X*: $x_{1},x_{2},\cdots ,x_{n}$, coefficients: $\beta_{0},\beta_{1},\cdots ,\beta_{n}$).

(1) $$P(Y=1)=\frac{1}{1+e^{-(\beta_{0}+\beta_{1}x_{1}+\beta_{2}x_{2}+\cdots +\beta_{n}x_{n})}}$$

(2) $$logit\;score=\beta_{0}+\beta_{1}x_{1}+\beta_{2}x_{2}+\cdots +\beta_{n}x_{n}$$

To separate the objective variable (SIRS-positive or negative) cases (logit score ≥ 0 suspected as SIRS-positive; logit score < 0 suspected as SIRS-negative), we developed a logistic regression as shown in Equation (3) for the M40FD-RR SIRS monitor using the M40FD-RR, HR, age, and sex as explanatory variables (Figure 1).

logit score = *β*_0_ + *β*_1_*M40FD-RR + *β*_2_*HR + *β*_3_*age + *β*_4_*sex (3)

### 2.2. Clinical Testing Method

#### 2.2.1. Patient Recruitment, Exclusion Criteria, and Nurse Records

This prospective observational study was conducted in accordance with the World Medical Association’s 1964 Declaration of Helsinki and approved by the Institutional Review Board of Suwa Central Hospital (Approval No. R2-6 (2020)). COVID-19-positive participants were recruited from patients admitted to Suwa Central Hospital from April 2020 to March 2021. The study included 29 patients (12 female, 17 male, aged 15–90 years) admitted to a general isolation ward dedicated to COVID-19 based on positive polymerase chain reaction testing [12]. Exclusion criteria included (1) death, discharge, or transfer to another hospital, (2) intake of prohibited drugs, and (3) when considered inappropriate by the investigator (Table 1). All patients were diagnosed based on the COVID-19 guidance criteria [13] established in Japan before 8 May 2023. Information including the patient’s age, sex, history of hospitalization, severity of COVID-19, comorbidities, and chronic diseases were all collected (Table 2). The daily nursing records obtained were infra-axillary temperature (thermometer), SpO2 (pulse oximeter), HR (pulse oximeter), blood pressure (sphygmomanometer), RR (visual counting), white blood cell count (WBC) derived by blood sampling, and PCR test result.

#### 2.2.2. Clinical Testing of the M40FD-RR SIRS Monitor

We conducted clinical testing of the M40FD-RR SIRS monitor, including daytime RR with artifacts induced by increased body movement. Nurses’ visual count of 60 s was used as a reference for RR (361 measurement points by nurses from 29 COVID-19 patients). Bland–Altman plots were used to compare M40FD-RR and RR, which were determined by conventional respiratory wave average peak duration for 90 s with RR determined by nurses’ visual counting.

In these 29 patients, we performed 109 evaluations of SIRS with blood sampling using the following criteria: body temperature > 38 °C or <36 °C, HR (assessed by the fingertip pulse sensor) > 90 bpm, RR (counted visually by a nurse/doctor) > 20 breaths per minute, and white blood cell count (WBC > 12,000 or <4000 cells/µL). The result was considered positive if two of these criteria were met.

#### 2.2.3. Sample Size and Outcome

The clinically acceptable limit of agreement (LOA) for the difference between the M40FD-RR and conventional measurements was defined as within ±4 BPM of RR and was determined within the 95% confidence interval (95%CI) [14,15]. The sample size of the patients in this study was designed to ensure that the M40FD-RR was comparable to the conventional method of measuring RR rate in COVID-19 patients, which is visual RR measurement by nurses. The sample size calculation method [10,14,15] was based on an expected difference of 4 BPM between the two groups, with a standard deviation of 2 BPM, a significance level of α: 0.05, and a statistical power of 1 − β of 0.9. As a result, the required sample size was 11 people, and data from 30 people were obtained to account for data loss. The reason for the large margin for data loss is that the variety of RR of COVID-19 patients was unknown in 2020 when the study was planned.

The sample size for logistic regression analysis was defined as 10 times the explanatory variable [16]. In this study, the data that were always obtained to separate SIRS positive and negative were four variables: RR, HR, sex, and age. If all were used, the number of explanatory variables would be 4, so the total number of samples was set to 40 or more. In addition, Leave-One-Out was adopted to obtain sufficient evaluation results in the small number of learning.

## 3. Results

### 3.1. Bland–Altman Plots of M40FD-RR and Conventional RR

**Figure 3:**
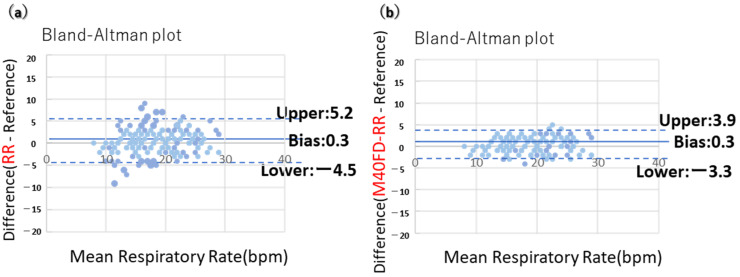
Bland–Altman plots of M40FD-RR and RR were determined by conventional respiratory wave average peak duration for 90 s using a piezoelectric respiratory sensor with RR determined by visual counting by a nurse. Bland–Altman plots of M40FD-RR showed markedly reduced error compared with conventional RR. (**a**) Conventional RR showed a bias of 0.3, 95% CI (−4.5 to 5.2); (**b**) M40FD-RR showed a bias of 0.3, 95% CI (−3.3 to 3.9).

Bland–Altman plots were used to assess the agreement between the two measurement methods. Bland–Altman plots of M40FD-RR showed markedly reduced error compared with conventional RR (Figure 3). Conventional RR showed a bias of 0.3, 95% CI (−4.5 to 5.2) (Figure 3a), while M40FD-RR showed a bias of 0.3, 95% CI (−3.3 to 3.9) (Figure 3b). The 95% CI for the M40FD-RR was within our defined clinically useful error range of ±4.

**Figure 4:**
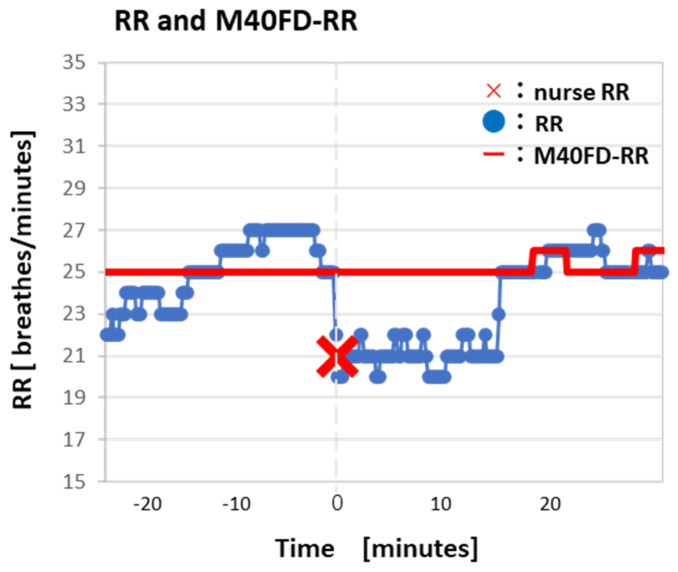
A case showing M40FD-RR drastically different from RR measured visually by a nurse (X).

Figure 4 shows a case where M40FD-RR is drastically different from RR, as measured visually by a nurse (X).

### 3.2. Classification Accuracy of the M40FD-RR SIRS Monitor

During the data collection period, subjects underwent blood tests, and 109 SIRS-positive and -negative (40 positive, 69 negative) results were obtained.

Negative and positive *t*-tests for each explanatory variable showed a significant difference (*p* < 0.01) only for HR.

The following logistic regression equation was derived:

logit score = −7.38 − 0.02M40FD-RR + 0.07HR + 0.04age − 0.42sex

A logit score ≥ 0 raises suspicion for SIRS, and a logit score < 0 indicates a low SIRS risk (Figure 1).

Without blood sampling data, clinical testing with the M40FD-RR SIRS monitor revealed 75% sensitivity and 83% negative predictive value.

**Figure 5:**
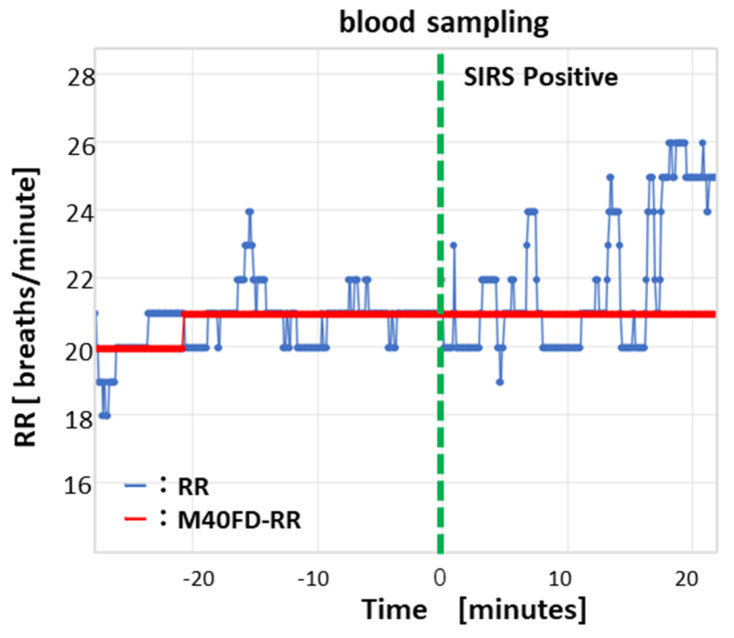
RR calculated using M40FD-RR rose from 20 to 21 breaths per minute 20 min before confirmation of SIRS using blood testing. Temporal variations in M40FD-RR (red line) and RR (blue line) were determined using the M40FD-RR SIRS monitor. Reference values of the SIRS inspection, including blood sampling, were as follows: WBC 3350 cells/µL, body temperature 38.8 °C, HR 88 bmp, and RR 22 breaths per minute (visually counted by a nurse).

In a SIRS-positive case determined with blood sampling (WBC 3350 cells/µL, body temperature 38.8 °C, HR 88 beats per minutes, RR (nurse) 22 BPM), the M40FD-RR calculated using approximately 500 RRs increased from 20 BPM to 21 BPM 20 min before SIRS testing with blood sampling (Figure 5). Conventional RR showed no apparent alteration before SIRS testing with blood sampling.

## 4. Discussion

Clinical testing of the M40FD-RR SIRS monitor achieved a sufficiently high sensitivity of 75% and a negative predictive value of 83%. The proposed non-contact M40FD-RR SIRS monitor places no burden on the patients by requiring neither blood sampling data nor any electrodes. Moreover, it can be used continuously for 24 h, so the system appears promising for preliminary SIRS monitoring.

The M40FD-RR monitor adopted in this system drastically improved the Bland–Altman plots compared with conventional RR for patients with mild to moderate severity COVID-19 who were equivalent to hospitalized patients in general wards except for being isolated. It is important to note that the patients included in this study had mild- to moderate-severity COVID-19, were hospitalized, and were conscious, meaning that their RRs fluctuated due to daily stressors. A limitation of the proposed systems is that M40FD-RR requires 40 min of measurement time, although artifacts induced by body movements were significantly reduced.

### 4.1. Promotion of Early Testing for SIRS and Reduction in Patient Burden

To promote early testing for SIRS by medical professionals, we have developed a preliminary SIRS monitor using vital signs, including M40FD-RR, that does not require blood sampling. In 2020, when the research plan was developed, COVID-19 required a special diagnostic method [11], but it is now treated the same as influenza and other infectious diseases. SIRS indices are indicators of inflammatory responses and are not contraindicated for infectious diseases, so we believe they can be used for other infectious diseases other than COVID-19. However, as shown in Table 2, the number of cases with other diseases was insufficient, so more data will need to be collected. In the future, we plan to evaluate the medical effectiveness of an improved version of the proposed system [17,18]. When the system achieves accuracy comparable to that of SIRS tests by medical professionals including blood sampling, regular blood tests for SIRS could be conducted only for patients determined to be possibly SIRS-positive by the system, thus reducing patient burden and medical costs.

### 4.2. Non-Contact HR Measurement to Reduce Patient Burden

The proposed system adopts a contact-type fingertip pulse oximeter. To reduce patient burden, it would be preferable to use a method for measuring HR that does not require patient contact. There are several methods for measuring HR that do not require contact [19], such as photoplethysmography [20] using a CMOS camera, Doppler radar using microwaves [21,22], and the piezoelectric sensor adopted in this study. So far, the piezoelectric sensor has not achieved equivalent accuracy to a contact-type fingertip pulse oximeter because the amplitude of body movements induced by the heartbeat is drastically smaller than that of respiration. A noise reduction algorithm under development for HR measurement using a piezoelectric sensor may be able to reduce patient burden in the future.

### 4.3. Reduction in the White Coat Effect by M40FD-RR

Figure 4 shows a case in which measurements made by M40FD-RR were drastically different from the RR measured visually by a nurse (X). Interestingly, the patient’s RR was in the range of 21 ± 1 for about 10 min, while a nurse performed tasks in the room. Because the patient was awake, we hypothesize that the nurse’s presence caused the patient to suppress their respiration rate either voluntarily or involuntarily. This suppression may have been caused by the white coat effect. Notably, the white coat effect induced by the nurse did not influence measurements taken via M40FD-RR.

### 4.4. Improvement of Accuracy Through the Addition of Prefrontal Cortex Temperature Measurement

Early changes in vital signs in SIRS induced by infection include changes in RR, HR, and body temperature. Our previously developed prefrontal cortex temperature measurement (PCTM) method [23] may improve the SIRS screening accuracy of the proposed system. The PCTM method enables the measurement of deep body temperature, i.e., prefrontal cortex temperature, using forehead temperature, which can be measured without contact and compared with the room temperature. In the future, the M40FD-RR SIRS monitor combined with the PCTM method may offer higher early SIRS screening accuracy.

### 4.5. Selecting the Best Sensor for RR

This study chose a piezoelectric sensor from among the various flexible pressure sensors available [24] because it is effective to place it under the mattress when measuring the target patients hospitalized with COVID-19. For this type of sensor, the environment should be one where electricity is constantly available and where only dynamic changes such as respiratory fluctuations will be captured. If the measurement is expanded to non-hospitalized COVID-19 or other infectious disease patients in the future, problems such as inability to measure under the mattress or power outages could arise. A self-powered flexible pressure sensor [24,25] may be able to solve the former problem.

## Figures and Tables

**Table 1:** Participants.

Consenting patients *	30
Excluded patients (%)	1 (3.3)
Patients with 1-day hospital stay (%) **	1 (3.3)
Number of participants (%)	29 (96.7)

* Number of patients who consented based on the consent form reviewed by the ethics committee; ** Hospitalization day one is excluded, as it does not comply with COVID-19 hospitalization rules because of a 3-day rest period after testing negative for COVID-19.

**Table 2:** Participant characteristics.

Number of Participants	N = 29
Mean age (year) [interquartile range (IQR)]	58.2 [15–90]
Sex, number (%)	
Male	17 (58.6)
Female	12 (41.4)
Mean hospitalization (days) [IQR]	9.0 [4–20]
COVID-19 severity, number (%)	
Severe	2 (6.9)
Moderate	12 (41.4)
Mild	14 (48.3)
No symptoms	1 (3.4)
Comorbidity, number (%)	
Pneumonia	16 (55.2)
Chronic disease, number (%)	
Hypertension	11 (37.9)
Dyslipidemia	4 (13.8)
Diabetes	3 (10.3)
Cardiac arrest	3 (10.3)
Fatty liver	2 (6.9)
Reflux esophagitis	2 (6.9)
Constipation	2 (6.9)
Bronchial asthma	2 (6.9)
Hyponatremia	1 (3.4)
Hypokalemia	1 (3.4)
Primary biliary cirrhosis	1 (3.4)
Hypothyroidism	1 (3.4)
Chronic liver disease	1 (3.4)
Hypercholesteremia	1 (3.4)
Hyperuricemia	1 (3.4)
Mitral valve replacement surgery	1 (3.4)
Atrial fibrillation	1 (3.4)
Gastric ulcer	1 (3.4)
Obesity	1 (3.4)
Liver failure	1 (3.4)
Aortic valve stenosis	1 (3.4)
Obstructive pulmonary disease	1 (3.4)
Thrombocytopenia	1 (3.4)
Leukopenia	1 (3.4)
Insomnia	1 (3.4)

## Data Availability

The data supporting the findings of this study are available on request from the Suwa Central Hospital. The data are not publicly available because they contain information that could compromise the privacy of research participants.
